# Green Tea Phenolic Epicatechins Inhibit Hepatitis C Virus Replication via Cycloxygenase-2 and Attenuate Virus-Induced Inflammation

**DOI:** 10.1371/journal.pone.0054466

**Published:** 2013-01-24

**Authors:** Ying-Ting Lin, Yu-Hsuan Wu, Chin-Kai Tseng, Chun-Kuang Lin, Wei-Chun Chen, Yao-Chin Hsu, Jin-Ching Lee

**Affiliations:** 1 Department of Biotechnology, College of Life Science, Kaohsiung Medical University, Kaohsiung, Taiwan; 2 Department of Chinese Medicine, ChiMei Medical Center, Tainan, Taiwan; 3 Graduate Institute of Natural Products, College of Pharmacy, Kaohsiung Medical University, Kaohsiung, Taiwan; University of Washington, United States of America

## Abstract

Chronic hepatitis C virus (HCV) infection is the leading risk factor for hepatocellular carcinoma (HCC) and chronic liver disease worldwide. Green tea, in addition to being consumed as a healthy beverage, contains phenolic catechins that have been used as medicinal substances. In the present study, we illustrated that the epicatechin isomers (+)-epicatechin and (−)-epicatechin concentration-dependently inhibited HCV replication at nontoxic concentrations by using *in vitro* cell-based HCV replicon and JFH-1 infectious systems. In addition to significantly suppressing virus-induced cyclooxygenase-2 (COX-2) expression, our results revealed that the anti-HCV activity of the epicatechin isomers occurred through the down-regulation of COX-2. Furthermore, both the epicatechin isomers additively inhibited HCV replication in combination with either interferon-α or viral enzyme inhibitors [2′-C-methylcytidine (NM-107) or telaprevir]. They also had prominent anti-inflammatory effects by inhibiting the gene expression of tumor necrosis factor (TNF)-α, interleukin (IL)-1β, and inducible nitrite oxide synthase as well as the COX-2 in viral protein-expressing hepatoma Huh-7 cells. Collectively, (+)-epicatechin and (−)-epicatechin may serve as therapeutic supplements for treating HCV-related diseases.

## Introduction

Hepatitis C virus (HCV) infection is a current global health problem, with an estimate of more than 170 million people chronically infected worldwide [1]. Chronic hepatitis associated with HCV infection increased the risk for progressive liver diseases, including fibrosis, cirrhosis, and hepatocellular carcinoma (HCC) [2]. No vaccine is currently available to prevent HCV infection. In addition, the severe side effects, including depression, fatigue, flu-like symptoms, and hemolytic anemia, of the current treatments with pegylated interferon-α (peg-IFN-α) plus ribavirin (RBV) often lead to treatment discontinuation [3]. More recently, two U.S. Food and Drug Administration (FDA) approval of the new-acting protease inhibitors, telaprevir and boceprevir, appear to be positive this regimen by triple therapy combined with peg-IFN-α/RBV, however, occurred side effects, such as anemia, and emergence of resistant variants limit the efficacy of these therapies [4]. Therefore, development of novel drugs or supplements for improving therapeutic efficacy of HCV-infected patients is still needed.

HCV is an enveloped virus that belongs to the *Hepacivirus* genus of the *Flaviviridae* family [5]. It has a 9.6-kb positive single-stranded RNA genome that comprises an open reading frame (ORF) and encodes a single polyprotein. The polyprotein is post-translationally processed by both the host and virus proteases into at least 10 mature individual proteins, including 4 structural proteins (C, E1, E2, and p7) and 6 nonstructural proteins (NS2, NS3, NS4A, NS4B, NS5A, and NS5B) [6]. NS5A is a serine phosphoprotein that promotes the inappropriate upregulation of many important risk factors for hepatocarcinogenesis, such as hepatic nuclear transcription factor-kappaB (NF-κB) and cyclooxygenase-2 (COX-2) [7], [8], [9]. COX-2 is an inducible COX isozyme that contributes to chronic inflammation and fibrosis through mediating the production of various prostaglandins (PGs). Some members of the PG family, such as PGE_2_, thromboxane B2, and prostacyclin, promote cellular proliferation, cancer invasiveness, angiogenesis, and anti-apoptosis [10], [11]. Many reports, including our previous studies, demonstrated that suppressing COX-2 protein levels could efficiently result in the suppression of HCV replication [12], [13], [14], [15]. Therefore, the interruption of COX-2 signaling is a potential approach for treating and chemopreventing HCV-related diseases by the coinstantaneous suppression of viral infection and hepatocarcinogenesis.

Green tea is produced from the leaves of the *Camellia sinensis*, which is widely consumed as a healthy beverage in China, Japan, and other Asian countries. Polyphenolic catechins (CATs) are the major active components of green tea, including (−)-epigallocatechin gallate (EGCG), (−)-epigallocatechin (EGC), (−)-epicatechin gallate (ECG) and four CAT isomers, (+)-CAT, (−)-CAT, (+)-epicatechin (EC) and (−)-EC, which possess various biological and pharmacological activities, including anti-inflammatory, antibacterial, antitumorigenic, and antiviral activities [16]. Among them, EGCG harbors a broad spectrum of antiviral activity against adenoviruses [17], Epstein–Barr virus [18], influenza virus [19], hepatitis B virus [20], and human immunodeficiency virus [21], herpes simplex virus 1 [22], and enterovirus 71 [23], although the intricate mechanisms of these antiviral properties remain to be elucidated. More recently, EGCG has been reported to exert anti-HCV activity by inhibiting viral entry [24], [25], viral NS3/4A protease [26], or NS5B polymerase [27]. To extend these findings, we evaluated whether the other polyphenolic CATs exert inhibitory effects on HCV replication and then investigated the molecular mechanism underlying these antiviral effects. Our data demonstrated that both the EC isomers, (+)-EC and (−)-EC, inhibit HCV replication by attenuating the COX-2-dependent signaling pathway. In addition, we further reveal that (+)-EC and (−)-EC could suppress the gene expression of HCV-induced pro-inflammatory enzymes and cytokines, such as TNF-α, IL-1β, inducible nitric oxide synthase (iNOS), and COX-2.

## Materials and Methods

### Cell culture and reagents

Human hepatoma cells (Huh-7) were cultured in Dulbecco's modified Eagle's medium (DMEM) with 10% heat-inactivated fetal bovine serum (FBS), 1% antibiotic–antimycotic solution, and 1% non-essential amino acids and were incubated at 37°C with 5% CO_2_ supplement. Ava5 cells (Huh-7 cells containing the subgenomic HCV genotype 1b replicon) [28] were cultured in DMEM with 10% heat-inactivated FBS, 1% antibiotic–antimycotic solution, 1% nonessential amino acids, and 1 mg/ml G418 and were incubated at 37°C with 5% CO_2_ supplement. The (+)-CAT, (−)-CAT, (+)-EC and (−)-EC with 98% purity were purchased from Kishida Chemical Co., Ltd, which were isolated from green tea leaves. The 2′-C-Methylcytidine (NM-107) and telaprevir was purchased from Toronto Research Chemicals Inc. and Legend Star International Co., Ltd, respectively. All tested compounds were stored at 10 mM in 100% dimethylsulfoxide (DMSO). The final concentration of DMSO in all reactions was maintained constantly at 0.1% in each experiment.

### Western blotting assay

Western blotting was performed as described previously [12]. The membranes were probed with specific antibodies, including anti-HCV NS5B, (1∶5000; Abcam, Cambridge, MA), anti-glyceraldehyde-3-phosphate dehydrogenase (GAPDH) (1∶10000; GeneTex, CA, USA), anti-Myc, anti-p65, anti-Lamin B (1∶1000; GeneTex), anti-phospho-ERK1/2, anti-phospho-p38, anti-phospho-JNK, anti-ERK1/2, anti-p38, anti-JNK monoclonal (1∶1000; Cell Signaling Technology, Inc. Danvers, MA, USA), or anti-COX-2 (1∶1000; Cayman, MI, USA) antibodies. The signal was detected using an ECL detection kit (PerkinElmer, CT, USA).

### Preparation of nuclear fraction

Nuclear extracts were prepared using NE-PER Nuclear and Cytoplasmic Extraction Reagents (Thermo Fisher Scientific Inc., USA) according to the manufacturer's instructions. Briefly, Ava5 cells were seeded in 6-cm dish at a density of 4×10^5^ cells/dish for 24 h and then were treated with or without EC isomers. After 3 days of incubation, nuclear extracts were prepared using the hypotonic buffer (10 mM HEPES, 1 mM MgCl_2_, 1 mM EDTA, 10 mM KCl, 0.5 mM DTT, 0.5% Nonidet P-40, 4 µg/ml leupeptin, 20 µg/ml aprotinin, and 0.2 mM PMSF). After centrifugation at 7000 g for 15 min, the pellets containing crude nuclei were resuspended in the hypertonic buffer (20 mM HEPES, 1.5 mM MgCl_2_, 0.2 mM EDTA, 0.6 M KCl, 0.5 mM DTT, 25% glycerol) at 4°C for 30 min. Finally, nuclear proteins were collected following centrifugation at 20000 g for 15 min and stored at −80°C until use.

### Quantitative real-time RT-PCR (qRT-PCR) analysis

Total cellular RNA was extracted using an RNA extraction kit (GMbiolab Co., Ltd, Taiwan) according to the manufacturer's instructions. The expression of HCV subgenomic RNA and cellular RNA was measured by quantitative real-time reverse-transcription polymerase chain reaction (qRT-PCR) analysis as previously described [12]. Each sample was normalized by the endogenous reference gene glyceraldehydes-3-phosphate dehydrogenase (*gapdh*). The cDNA quantification was performed by the ABI Step One Real-Time PCR-System (ABI Warrington, UK). The used primers were showed in Table 1.

**Table 1:** Oligonucleotide sequences for real-time RT-PCR.

Oligonucleotide name	Sequence 5′ – 3′
5′ NS5B	5′-GGA AAC CAA GCT GCC CAT CA
3′ NS5B	5′-CCT CCA CGG ATA GAA GTT TA
5′ GAPDH	5′-GTC TTC ACC ACC ATG GAG AA
3′ GAPDH	5′-ATG GCA TGG ACT GTG GTC AT
5′ TNF-α	5′-CCT GTG AGG AGG ACG AAC
3′ TNF-α	5′-AAG TGG TGG TCT TGT TGC
5′ IL-1β	5′-GGA GAA TGA CCT GAG CAC
3′ IL-1β	5′-GAC CAG ACA TCA CCA AGC
5′ iNOS	5′-CTT TGG TGC TGT ATT TCC
3′ iNOS	5′-TGT GAC CTC AGA TAA TGC
5′ COX-2	5′-CCG AGG TGT ATG TAT GAG
3′ COX-2	5′-TGG GTA AGT ATG TAG TGC

GAPDH, glyceraldehydes-3-phosphate dehydrogenase; TNF-α, tumor necrosis factor-α; IL-1β, interleukin-1β; iNOS, inducible nitric oxide synthase; COX-2, cyclooxygenase-2.

### HCV JFH-1 infection assay

Production of infectious HCV genotype 2a JFH-1 particles was transfected with *in vitro* transcribed full-length JFH-1 RNA into Huh-7.5 and the infectivity titer of JFH-1 was determined by immunostaining with anti-core antibody as described [29]. The inhibitory effect of each epicatechin on HCV infection was assayed as previously described [30]. In brief, the Huh-7 cells were seeded at density of 4×10^4^ cells/well in 24-wells culture plate and infected with 100 µl of HCV JFH-1 particles at a multiplicity of infection [31] of 0.02 for 6 h followed by incubation with various concentrations of EC isomers for an additional 72 h. Subsequently, total RNAs were collected and subjected to RT-qPCR for measuring mRNAs of HCV and GAPDH as described above.

### Cytotoxicity assay

Ava5 cells were seeded in 96-well plates at a density of 5×10^3^ per well and then incubated with compounds at various concentrations for 3 days. The cell viability was determined by the colorimetric 3-(4,5-dimethylthiazol-2-yl)-5- (3-carboxymethoxyphenyl)-2-(4-sulfophenyl)-2H-tetrazolium (MTS) assay (Promega Corporation, Madison, WI) as previously described [12].

### Analysis of the combination treatment

Ava5 cells were incubated with serially diluted EC isomers [(+)-EC or (−)-EC] at 50 and 75 µM in combination with diluted IFN-α (30 and 60 U/mL), NM-107 (0.75 and 1.5 µM) or telaprevir (0.3 and 0.6 µM) for 3 days. Total cellular RNA was harvested and analyzed using quantitative real-time qRT-qPCR, for which endogenous cellular *gapdh* expression served as an internal control.

### Transfection and luciferase activity assay

To assess COX-2 modulation by the EC isomers, the 1 µg of the reporter plasmid pCOX-2-Luc [12] was transfected into Ava5 cells using T-Pro reagent (Ji-Feng Biotechnology CO., Ltd. Taiwan) in accordance with the manufacturer's instructions. Six hours after transfection, the cells were incubated with different concentrations of the EC isomers for 3 days. To further investigate COX-2 regulation by the EC isomers, Ava5 cells were transfected with either control vector pcDNA4/*myc*-His-A (Life technologies, Carlsbad, CA) or various concentrations of COX-2 expression vector pCMV-COX-2-Myc (0.25–1.5 µg) in the presence of the EC isomers at 75 µM for 3 days. Cell extracts from each sample were prepared to measure luciferase activity using the Bright-Glo Luciferase Assay System (Promega) according to the manufacturer's protocol. To evaluate the role of COX-2 in HCV replication, Ava5 cells were transfected with increasing concentrations of the COX-2 shRNA expression vector (pCOX-2-shRNA; 0.5 to 2 µg) or LacZ shRNA, as a non-specific control. After 3 days of incubation, cell lysates were prepared for Western blotting with specific antibodies. To assess viral protein-induced inflammatory gene (TNF-α, IL-1β, iNOS and COX-2) modulation by the EC isomers, Huh-7 cells were transfected with 1 µg of pCMV-core-Myc or pCMV-NS5A-Myc in the presence of the EC isomers (50 and 75 µM). After 3 days of incubation, cell lysates were subjected to Western blotting with anti-Myc antibodies and cellular RNAs were subjected to qRT-PCR with specific primers (Table 1).

### Statistical analysis

The data were expressed as mean ± SD of at least three independent experiments. Statistical calculations were analysed by the Student's *t*-test; *p*-values <0.01 were considered statistically significant.

## Results

### Inhibitory effect of the CAT epimers on HCV replication

**Figure 1:**
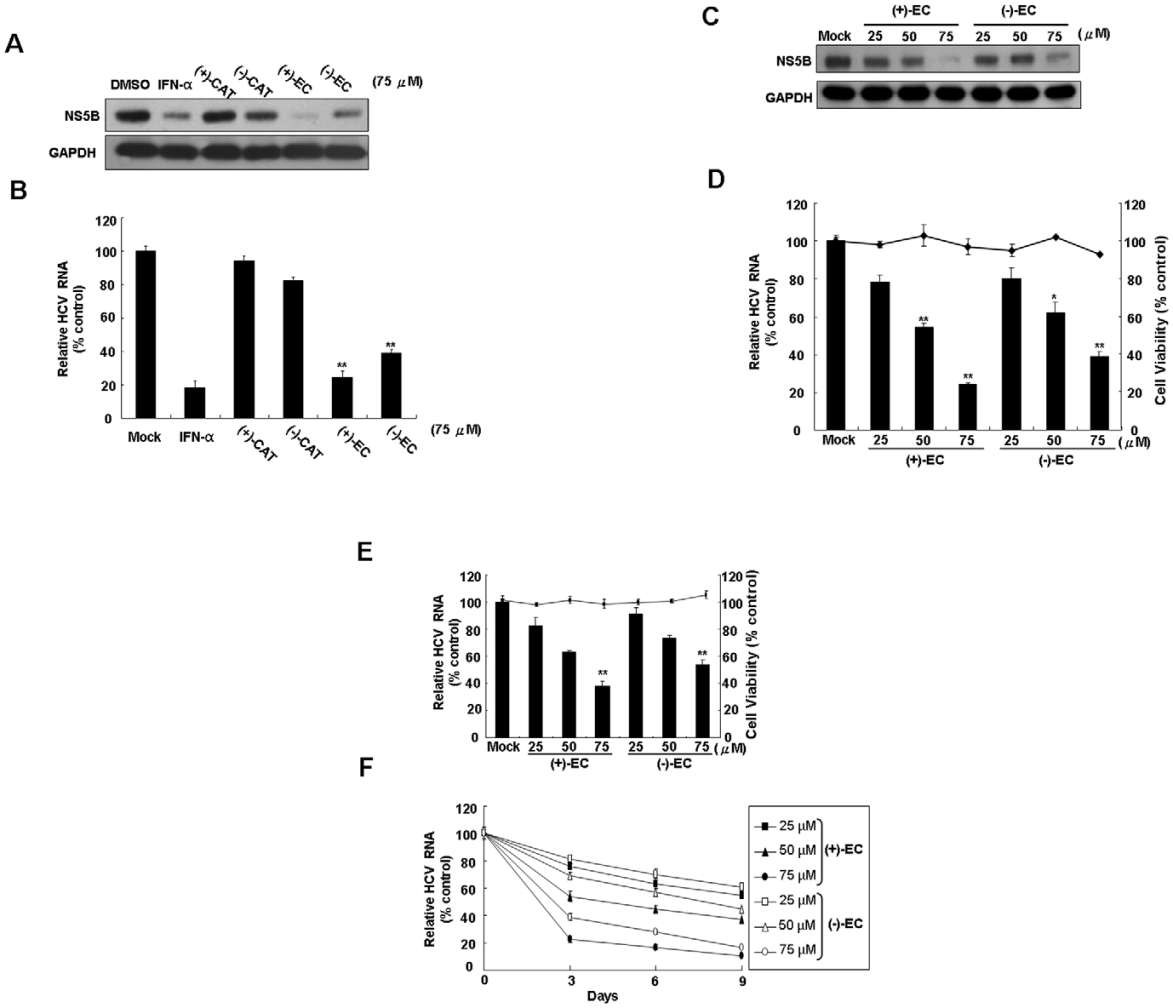
Anti-HCV activity of phenolic CATs. Inhibitory effect of various phenolic CATs on HCV (A) protein synthesis and (B) RNA replication. Concentration-dependent reduction of HCV (C) protein synthesis and (D) RNA replication in (+)-EC- or (−)-EC-treated HCV replicon cells. Ava5 cells were treated with each CAT isomers [(+)-CAT, (−)-CAT, (+)-EC and (−)-EC] at the indicated concentrations (25, 50, and 75 µM). After 3 days, the cell lysates were collected and then subjected to Western blotting with antibodies against NS5B and GAPDH (loading control). IFN-α (100 U/mL) and DMSO (0.1%) treatment served as the positive and mock controls, respectively. HCV RNA levels was quantified by qRT-PCR and normalized to *gapdh* mRNA levels after CAT isomers treatment for 3 days. Cellular toxicity was evaluated by the MTS assay after 3 days in the presence of the indicated concentrations of each EC isomers. (E) Concentration-dependent reduction of infectious HCV JFH-1 replication in (+)-EC- or (−)-EC-treated Huh-7.5 cells. After 6 h of JFH-1 virus incubation, Huh-7.5-infected cells were treated with EC isomers for 3 days. The levels of intracellular HCV RNA were determined by qRT-PCR following normalization of cellular *gapdh* mRNA. The efficacy of inhibition is expressed as the percentage relative to the RNA levels quantified without ECs (mock control). (F) Time-dependent reduction of HCV RNA levels in Ava5 cells treated with EC isomers. Ava5 cells were treated with EC isomers at concentration of 25, 50 and 75 µM. HCV RNA levels were quantified by qRT-PCR after EC isomers treatment for 3, 6 and 9 days. DMSO (0.1%) treatment served as the mock controls. Error bars represent the SD from three experiments. *P<0.05; **P<0.01.

To evaluate the inhibitory effects of CAT epimers on HCV protein synthesis, Ava5 cells were incubated with 75 µM of (+)-CAT, (−)-CAT, (+)-EC, and (−)-EC for 3 days, followed by Western blotting analysis. As shown in Fig. 1A, (+)-EC and (−)-EC treatment resulted in significant reductions in HCV protein levels compared with levels in the mock control (0.1% DMSO), in which IFN-α treatment served as a positive control. In contrast, (+)-CAT and (−)-CAT treatment exhibited insignificantly inhibited HCV protein synthesis at the tested concentration. To further determine the inhibitory effect of the CAT epimers on HCV replication, HCV RNA levels were determined by qRT-PCR. Similar results were observed, as (+)-EC and (−)-EC treatment resulted in a marked decrease in the HCV RNA levels (Fig. 1B). Next, to verify the antiviral activity of (+)-EC and (−)-EC, we treated Ava5 cells with various concentrations of (+)-EC and (−)-EC (25, 50 and 75 µM) and then performed Western blot and qRT-PCR analysises. As shown in Fig. 1C and D, (+)-EC and (−)-EC concentration-dependently inhibited HCV protein and RNA synthesis (left axis), respectively; slight cytotoxicity was observed at the effective concentrations (right axis). We further performed HCV JFH-1 infectious assay to confirm the anti-HCV activity of (+)-EC and (−)-EC (Fig. 1E, left axis), in which non-cytotoxicity was observed in JFH-1-infected Huh-7 cells at the assay conditions (right axis). To examine whether (+)-EC and (−)-EC can efficiently eliminate HCV replication, we treated Ava5 cells with increasing concentrations of EC isomers (25–75 µM) for a time-course incubation (0–9 days). qRT-PCR analysis revealed that HCV RNA levels were reduced by EC isomers in a time-dependent manner (Fig. 1F) with no effect on cell viability, as measured by the MTS assay (data not shown). The HCV RNA levels were largely eliminated after exposure to 75 µM EC isomers at 9 days, with an inhibition rate of 83.4–89.3%.

### ECs attenuate COX-2 expression in HCV replicon cells

**Figure 2:**
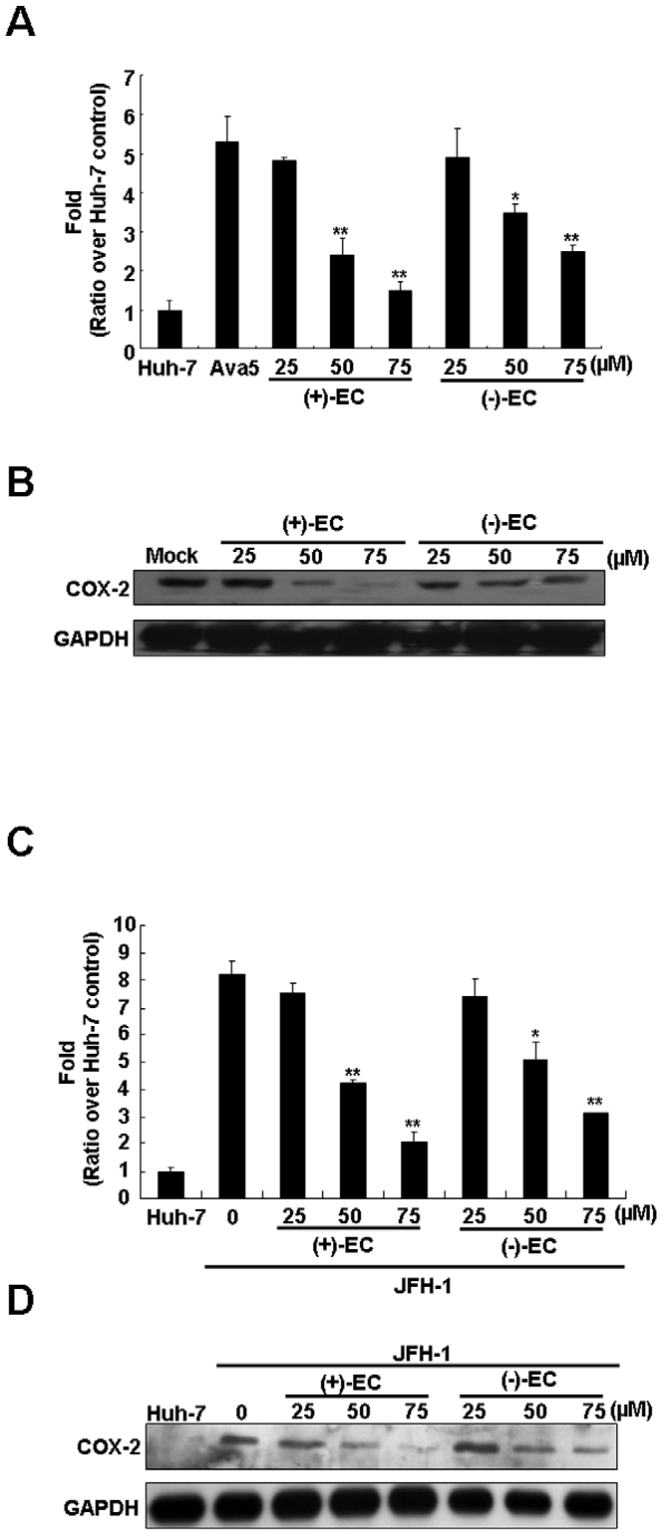
Inhibitory effect of the EC isomers on HCV-induced COX-2 expression. (A) The EC isomers reduce HCV-induced COX-2 gene promoter activity. Huh-7 cells or Ava5 were transfected with the pCOX-2-Luc reporter plasmid encoding firefly luciferase under control of the COX-2 promoter. After treatment with serial diluted concentrations of the EC isomers for 3 days, cell lysates were subjected to luciferase activity assays. The basal level of COX-2 promoter activity was identified in Huh-7 cells transfected with pCOX-2-Luc without EC isomers treatment, which is defined as 1 (B) The EC isomers reduce COX-2 protein expression. Ava5 cells were treated with the EC isomers at the indicated concentrations for 3 days. Cell lysates were subjected to Western blotting with anti-COX-2 and anti-GAPDH antibodies. (C) Concentration-dependent reduction of infectious HCV JFH-1-induced COX-2 gene promoter activity by EC isomers in Huh-7 cells. The pCOX-2-Luc transfected Huh-7 cells were incubated with JFH-1 virus for 6 h in the absence or presence of increasing concentrations of the EC isomers. After 3 days incubation, cell lysates were subjected to luciferase activity assays. (D) The EC isomers reduced COX-2 protein expression in JFH-1-infected Huh-7 cells. The JFH-1-infected Huh-7 cells were treated with the EC isomers at the indicated concentrations for 3 days. Cell lysates were subjected to Western blotting with anti-COX-2 and anti-GAPDH antibodies. Error bars represent the SD from three experiments. Asterisks indicate a significant difference compared with EC-untreated Ava5 or JFH-infected Huh-7 cells. *P<0.05; **P<0.01.

Multiple studies have illustrated that HCV proteins dramatically stimulate COX-2 expression, which is associated with carcinogenesis [9], [32]. To investigate whether the EC isomers could suppress HCV-induced COX-2 expression, we measured the transcription and translation levels of COX-2 in EC-treated Ava5 cells. The COX-2 promoter-linked luciferase reporter assay revealed that both (+)-EC and (−)-EC decreased luciferase activity in a concentration-dependent manner compared with the findings in 0.1% DMSO-treated Ava5 cells, and a significant effect was observed at 75 µM (Fig. 2A). As expected, COX-2 protein synthesis was suppressed by (+)-EC and (−)-EC treatment according to Western blot analysis (Fig. 2B). Next, we carried out the HCV JFH-1 infectious assay to confirm the suppression of HCV-induced COX-2 transcriptional activity and protein synthesis by (+)-EC and (−)-EC in a concentration-dependent manner (Fig. 2C and D). Based on these results, we suggest that the EC isomers attenuated COX-2 expression at the mRNA transcription level.

### The anti-HCV activity of ECs is mediated by the downregulation of COX-2 expression

**Figure 3:**
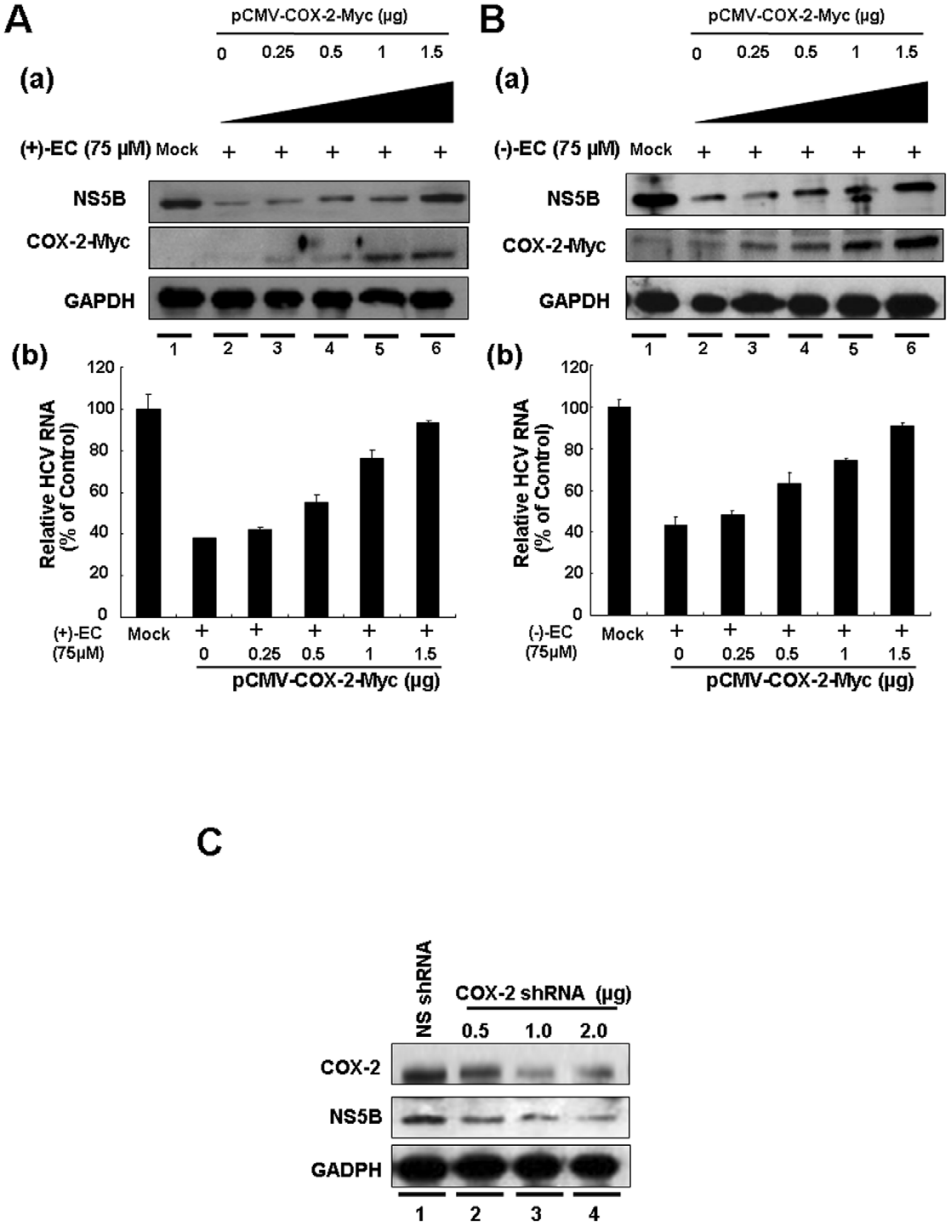
Concentration-dependent restoration of EC-reduced HCV protein synthesis and RNA replication by extraneous COX-2 expression. Ava5 cells were transfected with the indicated amounts of the COX-2 expression plasmid pCMV-COX-2-Myc encoding *cox-2* for 6 h, followed by either (A) (+)-EC or (B) (−)-EC treatment at a concentration of 75 µM for 3 days. “Mock” indicated transfection of control vector pcDNA4/*myc*-His-A in the presence of 0.1% DMSO. (a) Cell lysates were subjected to Western blotting with anti-NS5B, anti-COX-2, and anti-GAPDH antibodies to evaluate protein expression levels. (b) Total RNAs were subjected to qRT-PCR to evaluate HCV RNA levels. Error bars represent the SD from three experiments. (C) Reduction of HCV protein synthesis by COX-2 gene knockdown. Ava5 cells were transfected with either different amounts (0.5–2 µg) of the COX-2 shRNA or 2 µg of LacZ shRNA vectors as a control group. After 3 days of incubation, cell lysates were prepared for Western blotting with anti-COX-2 and anti-NS5B antibodies.

**Figure 4:**
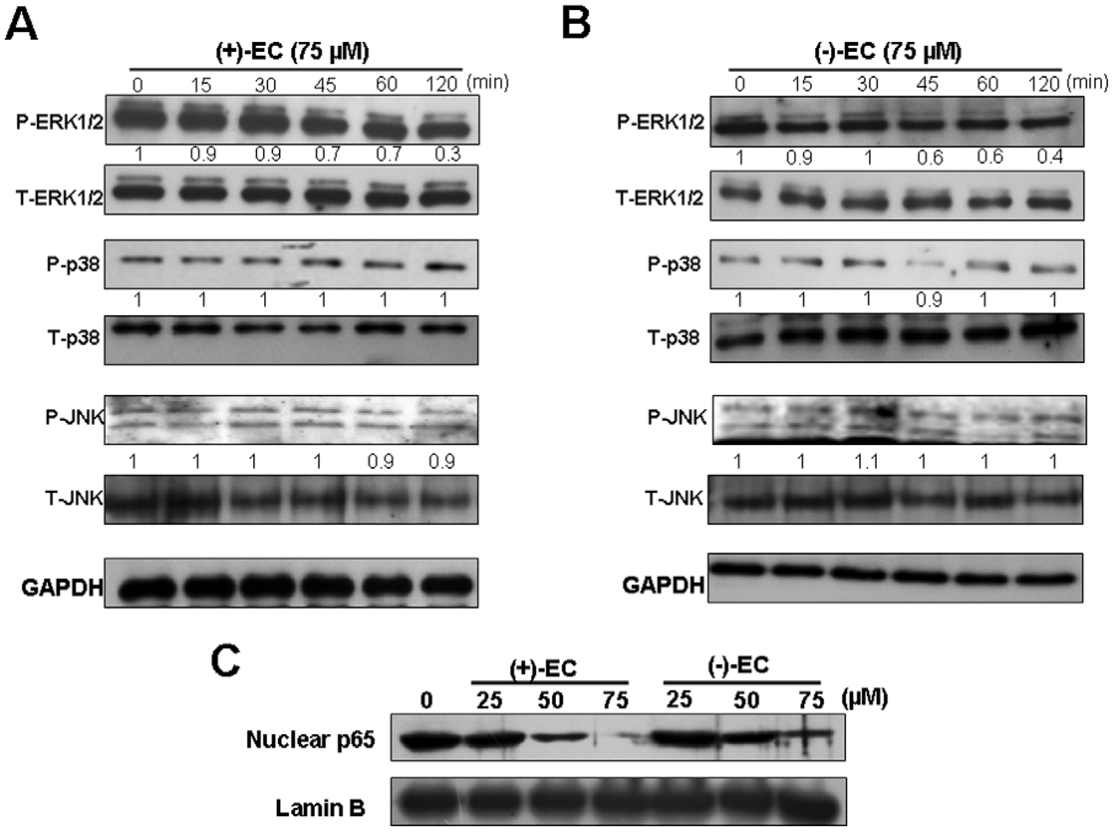
Inhibitory effect of the EC isomers on HCV-induced ERK and NF-κB pathways. Ava5 cells were treated with (+)-EC (A) and (−)-EC (B) at a concentration of 75 µM for indicated times (0, 15, 30, 45, 60, 120 min). Total cell lysates were prepared for Western blot analysis with specific antibody against P-ERK1/2, T-ERK, P-p38, T-p38, P-JNK, T-JNK, and GAPDH (loading control). (C) Ava5 cells were treated with the indicated concentrations of (+)-EC and (−)-EC for 3 days. The nuclear extracts were prepared for Western blot analysis with anti-p65 and anti-Lamin B. The relative blot intensities were quantified by densitometric scanning. The densitometry values were normalized to GAPDH and untreated values set as 1. Each experiment was performed in triplicate.

To further investigate whether COX-2 downregulation contributed to the inhibition of HCV replication by (+)-EC and (−)-EC, we used transient COX-2 overexpression and EC isomers treatment to assess their anti-HCV effects at the molecular level. Ava5 cells were transfected with either a control vector pcDNA4/*myc*-His-A or a pCMV-COX-2-Myc vector encoding the *cox-2* at various concentrations of transfected plasmid DNA (0.25, 0.5, 1, and 1.5 µg) and then treated with the EC isomers at 75 µM, a concentration that prominently inhibited HCV-induced COX-2 expression and HCV replication (Fig. 1 and 3). Western blotting analysis indicated that the gradual increase of exogenous COX-2-Myc expression [Fig. 3A(a) and 3B(a), middle panel, lanes 3–6] resulted in the recovery of HCV protein synthesis in the presence of the EC isomers (upper panel) compared with the findings in the control transfected cells in the absence or presence of the EC isomers (lanes 1 and 2). Consistent results were observed in qRT-PCR analysis, which revealed that COX-2-Myc overexpression momentously restored the EC-reduced HCV transcriptional levels in a concentration-dependent manner [Fig. 3A(b) and 3B(b)]. To clarify the effect of COX-2 reduction on HCV replication, we conducted gene silencing of COX-2 expression by transfection of COX-2 shRNA in Ava5 cells. The effect of COX-2 shRNA on COX-2 and HCV protein synthesis was examined by Western blotting. As shown in Fig. 3C, COX-2 shRNA concentration-dependently reduced COX-2 protein levels (upper panel, lanes 2–4), and simultaneously reduced HCV protein synthesis in a concentration-dependent manner (middle panel, lanes 2–4), whereas control shRNA had no effect on both COX-2 and HCV protein synthesis (lane 1). The inhibitory effect of COX-2 gene knockdown on the suppression of HCV protein synthesis was consistent with the results of ours [12], Trujio-Murillo et al. [15], Gretton et al. [13], and Okamoto et al. [14] by using selective COX-2 inhibitors. To further verify the upstream signaling mediators by which (+)-EC and (−)-EC downregulated HCV-induced COX-2 expression, we treated Ava5 cells with individual EC at 75 µM for 0–120 minutes and then examined the effect of the EC isomers on the phosphorylation of extracellular regulated protein kinase 1 and 2 (ERK1/2), p38 kinase, and c-Jun NH2-protein kinase (JNK), which are the major subfamily members of mitogen-activated protein kinase (MAPK) signaling pathway for COX-2 regulation [33]. Western blot analysis showed that the phospho-ERK protein levels were reduced by (+)-EC or (−)-EC treatment in a time-dependent manner following quantification of the immunoblots (Fig. 4A and 4B, upper panel, phospho-ERK 1/2 compared to total ERK1/2 following normalization of GAPDH). In contrast, phospho-p38 or phospho-JNK protein levels were insignificantly changed in EC-treated Ava5 cells, compared to the control (0 minute). In addition to MAPK signaling pathway, COX-2 is also mediated by NF-κB signaling pathway [34]. Therefore, we next examined the inhibitory effect of EC isomers on NF-κB activation. As shown in Fig. 4C, (+)-EC or (−)-EC treatment diminished amounts of nuclear NF-κB subunit p65 protein in a concentration-dependent manner (Fig. 4C), compared to the EC-untreated Ava5 cells. Taken together, these results supported our conclusion that COX-2 reduction was associated with the antiviral activity of the EC isomers.

### ECs additively inhibit HCV replication in combination with either IFN-α or viral enzyme inhibitors

**Figure 5:**
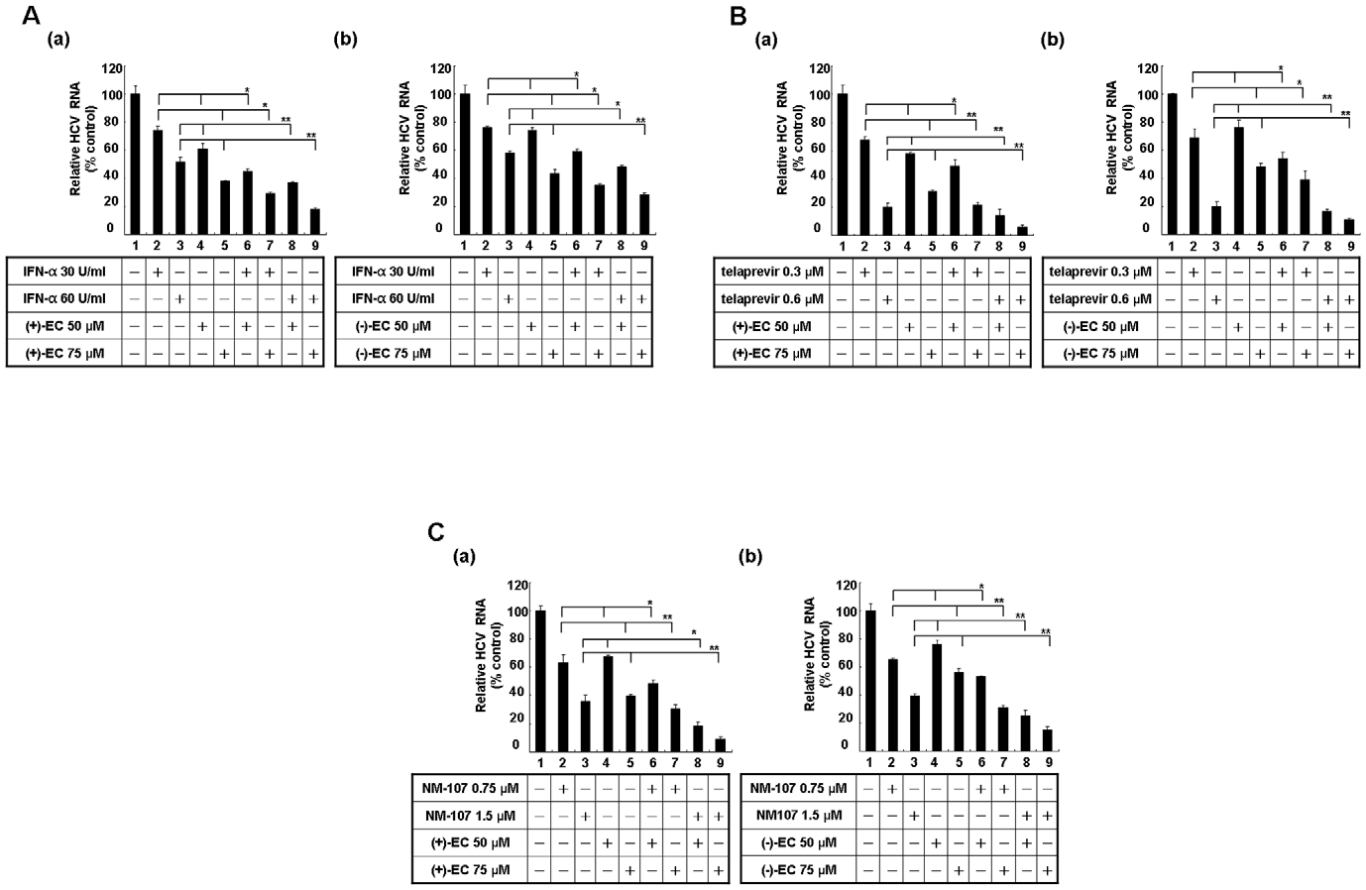
Effects of a combination of phenolic CATs and various inhibitors on HCV replication. Additive antiviral effect of the EC isomers combined with either (A) IFN-α or the viral enzyme inhibitors (B) telaprevir and (C) NM-107. Ava5 cells were treated with (a) (+)-EC or (b) (−)-EC in combination with each inhibitor at the indicated concentrations for 3 days. HCV RNA levels were quantified by qRT-PCR and normalized to *gapdh* mRNA levels. The efficacy of inhibition is expressed as the percentage relative to the RNA levels quantified without ECs. Error bars represent the SD from three experiments. Asterisks indicate a significant difference compared with single-compound treatment. *P<0.05; **P<0.01.

To investigate the potential therapeutic use in combinational regimens, HCV replicon cells were treated with ECs and either IFN-α, telaprevir (an NS3/4A protease inhibitor approved by the US Food and Drug Administration in May 2011) [35], or NM-107 (an NS5B polymerase inhibitor) [36] at fixed concentrations for 3 days. As shown in Fig. 5A, the combinations of the EC isomers with IFN-α resulted in enhanced inhibition of HCV replication (lanes 6–9) compared with the findings in samples incubated with each compound alone (lanes 2–5) or DMSO (lane 1). Similarly, combinations of the individual EC isomers with telaprevir [Fig. 5B(a) and (b)] or NM-107 [Fig. 5C(a) and (b)] also displayed an additive decrease in HCV RNA levels. These results indicated that the EC isomers may be promising adjuvant for anti-HCV therapy.

### ECs suppress the expression of pro-inflammatory mediators in HCV NS5A- and core-expressing cells

**Figure 6:**
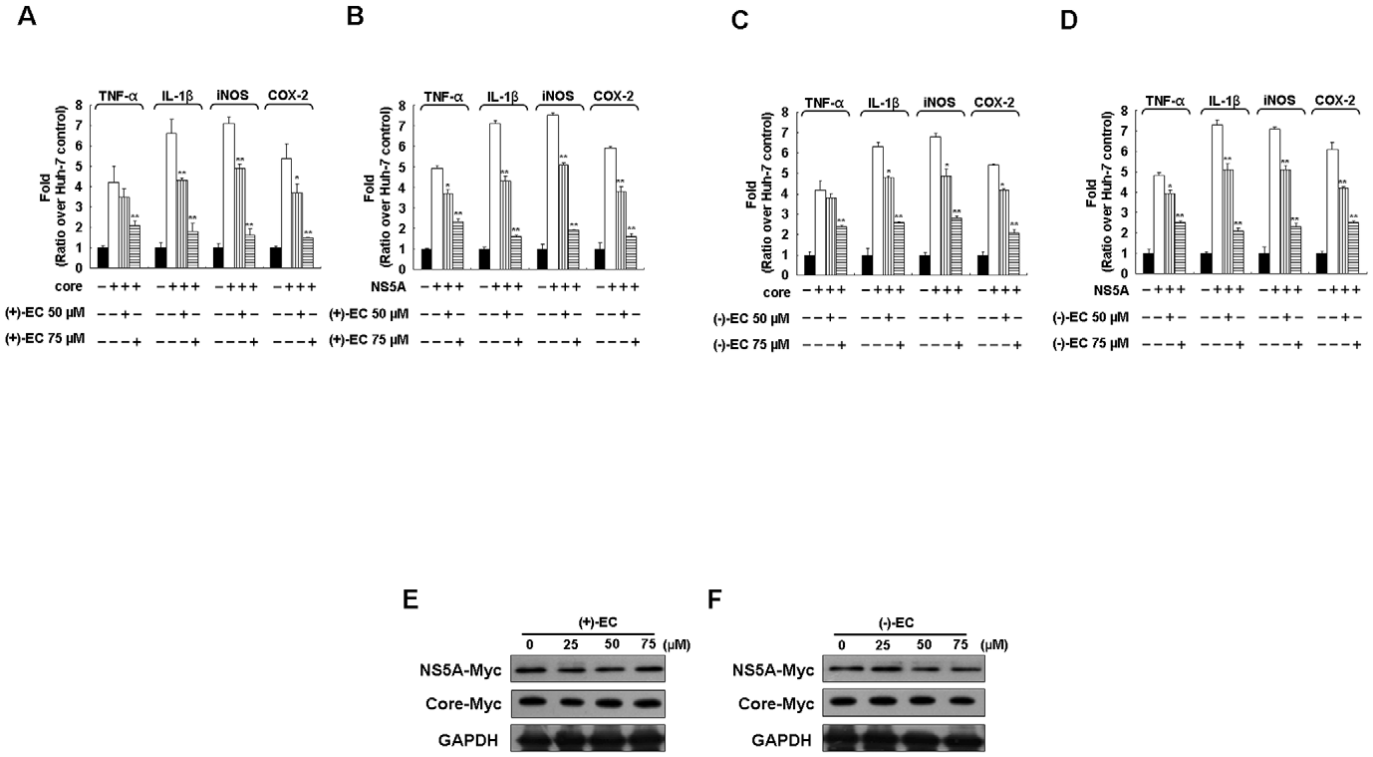
Inhibitory effect of the EC isomers on HCV core- and NS5A-induced inflammatory gene expression. Huh-7 cells were transfected with either the HCV core expression vector pCMV-core-Myc or the NS5A expression vector pCMV-NS5A-Myc in the presence of (+)-EC (A and B) or (−)-EC (C and D) at the indicated concentrations (50 and 75 µM) for 3 days. Total RNAs were subjected to qRT-PCR to evaluate the RNA levels of TNF-α, IL-1β, iNOS, and COX-2. Cell lysates of (+)-EC (E) or (−)-EC (F) were subjected to Western blotting with anti-Myc and anti-GAPDH antibodies. Error bars represent the SD from three experiments. Asterisks indicate a significant difference compared with EC-untreated core- or NA5A-transfected cells. *P<0.05; **P<0.01.

HCV proteins such as the structural core protein and the non-structural NS5A protein are well-known carcinogenic factors for HCV-related HCC through the induction of aberrant chronic inflammation [37]. Many pro-inflammatory gene products and cytokines, including TNF-α, IL-1β, COX-2, and iNOS, are indicated to be critical mediators of inflammatory diseases [38], [39]. To investigate the hepatoprotective effect of the EC isomers against core- and NS5A-induced pro-inflammatory gene expression as described above, Huh-7 cells transiently overexpressing either core or NS5A were incubated with various concentrations of the EC isomers (50 and 75 µM) for 3 days. qRT-PCR analysis illustrated that the increased mRNA levels of those stimulated pro-inflammatory mediators were concentration-dependently decreased by the EC isomers, as compared to the findings in untreated cells (Fig. 6A–D). To examine whether the attenuation of viral protein-induced pro-inflammatory gene expression by EC isomers may be due to the reduction of viral proteins over time in a transient transfection assay, we performed Western blotting to detect the levels of both Myc-tagged core and NS5A proteins using anti-Myc antibody. As shown in Fig. 6E and F, similar amounts of core or NS5A protein expression were observed in the absence or presence of an increased concentration of each EC isomer at 3 days post-transfection, which excluded the time effect of transient decrease in viral protein expression in anti-inflammatory analysis.

## Discussion

Green tea is widely consumed as a healthy beverage, and it has been considered as an alternative medicine for preventing cancer formation and infectious diseases owing to its diverse pharmacological effects [40]. In the present study, we clearly demonstrated that both EC optical isomers [(+)-EC and (−)-EC] isolated from green tea leaves exert inhibitory effects on HCV replication at both protein and RNA levels without causing host cellular toxicity (Fig. 1). This result was consistent with those reported by Takeshita *et al,* who demonstrated that EC isolated from blueberry leaves displayed anti-HCV activity as assessed using an HCV subgenomic replicon (genotype 1b, HCV-Con1), with an EC_50_ of 92 µM [41], which is similar to our finding in HCV subgenomic replicon (genotype 1b, HCV-Con1) and JFH-1 (genotype 2a) infectious systems. However, this observation was differed from a report by Calland *et al*, who found that (−)-EC isolated from organic Japanese tea had no anti-HCV activity at 50 µM by using an infectious recombinant HCV expressing the *Renilla* luciferase (genotype 2a, JFH1-Luc) [42]. These discrepant results may be attributable to differences in the assay methods used, or concentration tested. Li *et al* previously reported that procyanidin B1, a dimer complex of (+)-CAT and (−)-EC isolated from *Cinnamomi* cortex, could suppress HCV replication. In contrast, (+)-catechin or (−)-EC alone did not inhibit HCV replication [43]. Therefore, the phenolic CATs isolated from different sources may contain different combining structures or purity, possibly leading to the divergent results obtained by different groups. In the case of polyphenolic EGCG, numerous studies recently demonstrated the different mechanisms of action regarding its inhibitory effects on HCV infection and replication, such as the inhibition of viral entry [42], NS3/4A protease [26], or NS5B polymerase [27]. More tests, such as characterization of the geometric structures of extracted compounds by comparison of the nucleophilic, electrophilic or radical frontier density, are required to distinguish the antiviral effects of green tea constituents and develop more potential derivatives of catechin against HCV infection.

In recent years, many groups have successfully developed direct-acting antivirals (DAAs) against HCV infection [44]. However, the efficacy of DAAs will be limited because they rapidly induce the emergence of HCV variants that are resistant to DAA treatment [45]. Additionally, different HCV genotypes also affect the viral sensitivity of virus to these compounds. Accordingly, targeted inhibition of host factors critical for HCV infection has been suggested as a promising strategy to escape viral resistance and induce drug susceptibility for a broad range of HCV genotypes [46]. Recent studies by us and others have proposed that suppressing HCV-stimulated COX-2 expression plays a critical role in inhibiting HCV replication [12], [13], [14], [15]. In the present study, we clearly demonstrated that (+)-EC and (−)-EC could effectively suppress HCV-mediated COX-2 production at the transcriptional level (Fig. 2A and 2C), and this suppression clearly illustrated the molecular mechanism underlying the anti-HCV activity of both the EC isomers through mediation of ERK-MAPK and NF-κB signaling pathways for COX-2 reduction (Figs. 3 and 4). We further investigated whether EC isomers inhibited HCV replication by targeting viral entry, internal ribosome entry site (IRES) translation, NS3/4A protease activity, NS5B polymerase activity, or viral secretion using reporter assay systems and JFH-1 infectious system previously established [47], [48], [49], [50]. The results indicated that there was no significant inhibition on the above targets by EC isomers treatment (Fig. S1). Collectively, this report provides the first evidence that EC isomers could block HCV replication by targeted inhibition of host cell factors required for the viral life cycle, which may improve the therapeutic success rate by overcoming drug resistance because of lower genetic mutation in host genome compared to the RNA virus genome [51]. Multiple therapeutic regimens involving peg-IFN-α/RBV in combination with small molecules that target distinct factors of the HCV life cycle appear to be promising therapeutic approaches. Our results revealed an additive effect of the EC isomers in combination with either IFN-α or viral enzyme inhibitors on inhibition of HCV replication (Fig. 4), revealing that (+)-EC and (−)-EC may serve as therapeutic supplements in peg-IFN-α/RBV or peg-IFN-α/RBV-free regimens. Recently, a defined green tea extract (GTC), Veregen®, containing polyphenolic catechins, was approved as an new drug application by U.S. FDA in 2006 and was commercially available for tropical treatment against genital and perianal warts [52]. Because of acceptable biosafety and bioavailability at regulated conditions, various types of standardized GTC with different ratio of polyphenolic catechins were recently used to explore their chemopreventive effect against cancer in clinical trial [53], [54], [55]. Therefore, a mixture of catechins may be potentially developed as a standardized pharmaceutical form or dietary supplement for prevention of HCV-related diseases in the future.

HCV core and NS5A function as etiological proteins that significantly induce inflammatory factors to initiate and maintain cancer cell survival and growth [2]. Our results demonstrated that HCV core and NS5A greatly stimulated pro-inflammatory gene activation, whereas a gradual suppression of COX-2, iNOS, TNF-α, and IL-1β RNA expression levels was observed upon either (+)-EC or (−)-EC treatment in HCV core- and NS5A-expressing cells, as shown in Fig. 5. Therefore, (+)-EC and (−)-EC may be useful as potential dietary supplements in the prevention and treatment of chronic HCV infection by simultaneous inhibition of viral replication, inflammation and virus-induced carcinogenesis. In conclusion, green tea (+)-EC and (−)-EC protect against both HCV replication and virus-induced inflammation. Because green tea is one of the most popular beverage and dietary supplements, it will provide beneficial effects for preventing or treating HCV-linked liver diseases.

## Supporting Information

Figure S1 **The effect of the EC isomers on HCV entry, assembly, NS3 protease, NS5B RdRp and IRES activity.** (A) Effect of EC isomers HCV JFH1 entry. Huh7.5 cells were seeded at a density of 4×10^4^ cells per well in 24-well plates were pre-incubated with indicated concentrations of EC isomers (50 and 75 µM), EGCG (50 µM), or anti-CD81 (α-CD81; 10 µg/ml, as a positive control) for 1 h and then were infected with HCV JFH-1 at an MOI of 0.02 for 6 h in the presence of the inhibitor. After 3 days, HCV RNA levels were quantified by qRT-PCR and normalized to *gapdh* mRNA levels. (B) Effect of the secretion of HCV JFH-1 by EC isomers. Huh-7.5 cells were seeded at a density of 4×10^4^ cells per well in 24-well plates. After 6 h of JFH-1 virus incubation, the virus-infected cells were treated with HCV secretion inhibitor naringenin (150 µM) or at indicated concentrations of EC isomers. After 3 days incubation, supernatants containing secreted JFH-1 cells were collected and the infectivity titer was determined by infecting Huh-7.5 cells. and HCV-infected cells were cultured further. Five days postinfection, HCV RNA levels were quantified by qRT-PCR. (C) Effect of the EC isomers on HCV NS3/4A protease activity. Huh-7 cells were co-transfected with 0.5 µg of the reporter plasmid [pEG(DEΔ4AB)SEAP] and the HCV NS3/4A expression vector pCMV-NS3/4A-Myc for 6 h and then treated with EC isomers at a concentration of 50 or 75 µM for 3 days. Culture medium was collected and subjected to measurement of secreted alkaline phosphatase (SEAP) activities by using Phospha-Light assay kit (Tropix, Foster City, CA, USA). Treatment with 10 µM of specific NS3/4A inhibitor telaprevir served as a positive control. (D) Effect of the EC isomers on HCV NS5B polymerase activity. Huh-7 cells were co-transfected with the 0.5 µg of reporter plasmid (p(+)FLuc-(−)UTR-RLuc) and HCV NS5B expression vector pCMV-NS5B-Myc for 6 h and then treated with EC isomers at a concentration of 50 or 75 µM for 3 days. The cells lysates were subjected to luminescence detection with the Dual-Glo Luciferase Assay Kit (Promega). Treatment with 0.3 µM of specific NS5B inhibitor PSI-7977 served as a positive control. (E) Effect of the EC isomers on HCV IRES activity. Huh-7 cells were transfected with 0.5 µg of the HCV IRES reporter (pFLuc-UTRΔC-RLuc) for 6 h and then treated with EC isomers at a concentration of 50 or 75 µM for 3 days. Total cells lysates were subjected to luminescence detection with the Dual-Glo Luciferase Assay Kit (Promega). Each value represents the mean ± SD of triplicate experiments after normalization of luciferase activities. *P<0.05; ** *P*<0.01.
(TIF)
