# Supplementary figures and images for: Green Tea Phenolic Epicatechins Inhibit Hepatitis C Virus Replication via Cycloxygenase-2 and Attenuate Virus-Induced Inflammation
Source: PLoS One. 2013 Jan 24;8(1):e54466. doi: 10.1371/journal.pone.0054466 (PMC3554764; doi:10.1371/journal.pone.0054466)

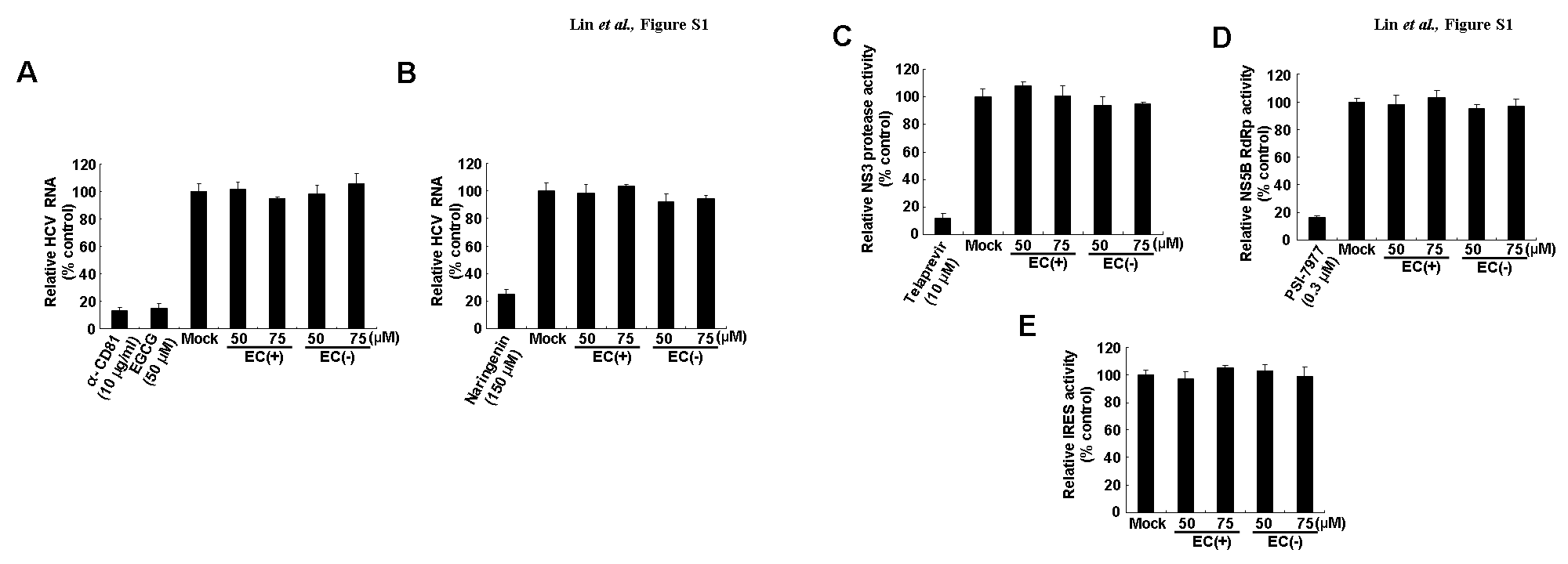

Supplement: Figure S1 — The effect of the EC isomers on HCV entry, assembly, NS3 protease, NS5B RdRp and IRES activity. (A) Effect of EC isomers HCV JFH1 entry. Huh7.5 cells were seeded at a density of 4×10^4^ cells per well in 24-well plates were pre-incubated with indicated concentrations of EC isomers (50 and 75 µM), EGCG (50 µM), or anti-CD81 (α-CD81; 10 µg/ml, as a positive control) for 1 h and then were infected with HCV JFH-1 at an MOI of 0.02 for 6 h in the presence of the inhibitor. After 3 days, HCV RNA levels were quantified by qRT-PCR and normalized to gapdh mRNA levels. (B) Effect of the secretion of HCV JFH-1 by EC isomers. Huh-7.5 cells were seeded at a density of 4×10^4^ cells per well in 24-well plates. After 6 h of JFH-1 virus incubation, the virus-infected cells were treated with HCV secretion inhibitor naringenin (150 µM) or at indicated concentrations of EC isomers. After 3 days incubation, supernatants containing secreted JFH-1 cells were collected and the infectivity titer was determined by infecting Huh-7.5 cells. and HCV-infected cells were cultured further. Five days postinfection, HCV RNA levels were quantified by qRT-PCR. (C) Effect of the EC isomers on HCV NS3/4A protease activity. Huh-7 cells were co-transfected with 0.5 µg of the reporter plasmid [pEG(DEΔ4AB)SEAP] and the HCV NS3/4A expression vector pCMV-NS3/4A-Myc for 6 h and then treated with EC isomers at a concentration of 50 or 75 µM for 3 days. Culture medium was collected and subjected to measurement of secreted alkaline phosphatase (SEAP) activities by using Phospha-Light assay kit (Tropix, Foster City, CA, USA). Treatment with 10 µM of specific NS3/4A inhibitor telaprevir served as a positive control. (D) Effect of the EC isomers on HCV NS5B polymerase activity. Huh-7 cells were co-transfected with the 0.5 µg of reporter plasmid (p(+)FLuc-(−)UTR-RLuc) and HCV NS5B expression vector pCMV-NS5B-Myc for 6 h and then treated with EC isomers at a concentration of 50 or 75 µM for 3 days. The cells lysates were [file pone.0054466.s001.tif]
